# Endometriosis at the Molecular Crossroads: Pathways, Targets, and Emerging Therapeutic Strategies

**DOI:** 10.3390/cimb48101001

**Published:** 2026-09-29

**Authors:** Jakub Toczek, Kasia Major, Rafał Stojko, Marcin Sadłocha

**Affiliations:** 1 Chair and Department of Gynecology, Obstetrics and Oncological Gynecology, Medical University of Silesia in Katowice, Markiefki 87, 40-211 Katowice, Polandmsadlocha@sum.edu.pl (M.S.); 2 Chair and Department of Pediatrics and Neonatology Unit, Clinical Hospital for Children in Chorzów, Władysława Truchana 7, 41-500 Chorzów, Poland

**Keywords:** endometriosis, progesterone resistance, NF-κB, angiogenesis, epigenetics, hypoxia, PI3K/AKT/mTOR signaling, biomarkers, precision medicine, therapeutic targets

## Abstract

Endometriosis affects an estimated one in ten women of reproductive age and remains defined clinically by the presence of endometrial-like tissue outside the uterine cavity, yet its persistence depends on biological processes that extend well beyond estrogen exposure. This review argues that endometriosis is best understood as a set of molecularly heterogeneous, interconnected disease processes, hormonal, immune, vascular, epigenetic, and metabolic, that converge on a compact set of intracellular signaling nodes rather than as a single hormonal disorder. These convergence points are mapped onto candidate therapeutic targets spanning established, investigational, and preclinical stages of development, and onto biomarker strategies organized here by clinical application, diagnosis, disease stratification, prognosis, and treatment monitoring, rather than by sample source alone. Translational obstacles, including the limited predictive value of current animal models, the recurring disconnect between preclinical and clinical results, and the distinction between symptom control and disease modification, are addressed throughout rather than confined to a single closing section. Endometriosis management continues to rely on symptomatic hormonal suppression; treatment strategies grounded in the disease’s molecular heterogeneity, rather than a uniform hormonal model, appear to offer the most realistic path toward durable, individualized management.

## 1. Introduction

Endometriosis is conventionally described as a chronic, estrogen-dependent, inflammatory condition defined by the presence of endometrial-like glands and stroma outside the uterine cavity. It affects an estimated one in ten women of reproductive age [1,2] and remains a persistently frustrating diagnosis in gynecology, not because its existence is in doubt, but because the gap between what clinicians can offer and what patients need has closed only slowly over several decades of research. Standard management, built around ovulation suppression, progestin exposure, or surgical excision, treats the disease as if a single hormonal switch were responsible for its persistence. In practice, recurrence after surgery is common, symptom control is partial for a meaningful share of patients, and the clinical picture, ranging from silent lesions found incidentally to severe pelvic pain and infertility, does not map neatly onto lesion size or hormonal exposure alone.

The last fifteen years of laboratory work have pushed the field toward a different picture. Estrogen dependence is real, but it is layered onto a local inflammatory environment that behaves more like a self-sustaining circuit than a downstream consequence of hormone exposure. Immune cells recruited to the peritoneal cavity fail to clear ectopic tissue and instead appear to support its survival [3,4]. The lesions acquire their own blood supply and nerve fibers [5], which plausibly explains why pain intensity in some patients bears little relationship to what is visible at laparoscopy. Chromatin marks and non-coding RNAs shift in ways that make some of these changes heritable at the cellular level, potentially explaining why lesions persist even after their initiating hormonal stimulus is suppressed [6]. Ectopic endometrial cells also adopt a metabolic profile more familiar from tumor biology than from normal endometrium, favoring glycolysis and tolerating hypoxia in a manner that supports growth within a hostile peritoneal microenvironment [7].

None of these mechanisms operates in isolation. Hormonal signaling shapes the local immune environment; the immune environment sustains angiogenesis and nerve growth; angiogenesis and hypoxia reprogram cellular metabolism; and epigenetic changes lock several of these adaptations in place.

Genetic predisposition, reproducible-if-individually-modest risk loci near WNT4, GREB1, and VEZT, and environmental exposure to endocrine-disrupting chemicals such as bisphenol A and phthalates add a further layer of susceptibility that narrows the population in which this cascade can take hold before a single lesion has formed [8,9,10]. General clinical overviews of endometriosis already exist [11,12]; this review adds a narrower, explicitly integrative argument organized around four questions that recur throughout the sections that follow: where the hormonal, immune, vascular, epigenetic, and metabolic axes converge on shared intracellular signaling nodes; which of the resulting targets show translational potential beyond an attractive preclinical rationale; which molecular features could support matching a patient’s disease to a specific treatment rather than a uniform hormonal regimen; and which mechanisms plausibly explain partial treatment response and recurrence. The distinction between mechanistic promise and clinical validation, and between symptom control and disease modification, is addressed throughout rather than reserved for a single closing section.

### Literature Search Strategy

This review follows a narrative rather than a systematic format, given the breadth of mechanisms and therapeutic targets it covers; a single PRISMA-style protocol across every subtopic addressed here would not meaningfully standardize a search this broad, but methodological transparency is still necessary. Literature was identified through PubMed/MEDLINE, Scopus, and Google Scholar, covering publications from January 2016 through August 2026 to prioritize current evidence, particularly for the epigenetic, microRNA, and targeted-therapy literature, where the field has moved quickly. Six classical, foundational studies published before 2016 are retained outside this window because they established a mechanism still directly cited in current endometriosis research, and each is identified as such at first citation in the text: the genome-wide association study identifying the WNT4, GREB1, and VEZT risk loci discussed in Section 1 (Rahmioglu et al., 2014) [9]; the original description of the HIF-1α/HIF-2α “HIF switch” discussed in Section 5 (Koh & Powis, 2012) [13]; let-7-mediated repression of RAS, discussed in Section 6 (Johnson et al., 2005) [14]; miR-200-family repression of ZEB1/ZEB2, discussed in Section 6 (Gregory et al., 2008) [15]; the MeCP2–histone deacetylase corepressor mechanism, discussed in Section 6 (Nan et al., 1998) [16]; and the foundational review of histone modification biology, discussed in Section 6 (Bannister & Kouzarides, 2011) [17]. No other reference in this review predates 2016. Searches combined the term “endometriosis” with mechanism-specific keywords, including but not limited to “progesterone resistance”, “NF-κB”, “JAK/STAT”, “PI3K/AKT/mTOR”, “angiogenesis”, “HIF”, “epigenetics”, “microRNA”, “biomarker”, and “therapeutic target”, restricted to English-language publications. Primary research articles, systematic reviews, and meta-analyses were preferred over narrative reviews when both were available on a given point; narrative reviews were used mainly for contextual framing and, where possible, traced back to the primary studies they cite. Case reports, non-peer-reviewed preprints, and conference abstracts were excluded. Reference lists of key reviews and included primary studies were hand-searched for additional citations not captured by the keyword search. During manuscript preparation, an AI language model (Claude, Anthropic) assisted with literature search, initial screening, and reference formatting; every AI-suggested reference was independently retrieved and checked by the authors against the original publication (title, author list, journal, year, and DOI) before inclusion, and the mechanistic and clinical interpretations in the text reflect the authors’ own reading of the primary sources. Where primary studies reported discordant findings for a given mechanism, both results are noted explicitly in the text rather than resolved through selective citation, and greater weight is given to findings replicated across more than one independent research group or corroborated by convergent human tissue and preclinical data; single-study findings are identified as such where this distinction matters for interpretation. Studies were further categorized by the type of evidence they provide, in vitro, animal model, human correlative or tissue-based, or human interventional, using the four-tier classification introduced in Section 8 (Table 1) and applied consistently to the therapeutic strategies discussed in Section 9 (Table 2), so that mechanistic plausibility is not conflated with clinical applicability.

## 2. Pathobiology of Endometriosis

The dominant explanatory framework for how endometriosis begins remains Sampson’s retrograde menstruation theory: endometrial fragments shed during menstruation travel backward through the fallopian tubes into the peritoneal cavity, where they attach to peritoneal or ovarian surfaces instead of being cleared. Retrograde menstruation itself is close to a universal phenomenon among menstruating women, which raises the obvious question of why only a subset go on to develop disease [1,3]. The answer, as far as current evidence allows, lies less in the act of ectopic seeding and more in what happens afterward: whether the shed tissue survives, attaches, invades, and builds the vascular and immune support it needs to persist.

Survival begins with adhesion. Endometrial fragments that reach the peritoneal surface must attach to mesothelium before local defenses can act, and altered expression of adhesion molecules and integrins on both the endometrial fragments and the peritoneal lining appears to favor this step in women who develop disease. Once attached, invasion follows a pattern that borrows heavily from the language of tumor biology: matrix metalloproteinases degrade the extracellular matrix and basement membrane, allowing ectopic cells to penetrate the peritoneal surface and establish a foothold below it rather than remaining a superficial deposit that could be cleared more easily [2].

Persistence depends on two further processes that unfold over a longer timescale. The first is angiogenesis: a lesion without its own blood supply is limited to what it can obtain by diffusion, which caps its growth and survival prospects, so ectopic endometrial cells respond by upregulating angiogenic factors, most notably vascular endothelial growth factor (VEGF), establishing a microvasculature adequate to sustain an expanding lesion [5,18]. The second is immune evasion. Peritoneal macrophages are normally expected to phagocytose and clear ectopic endometrial debris, much as they do in women without disease. In endometriosis, this surveillance function appears to fail: macrophages recruited to the lesion site are skewed toward a phenotype that promotes tissue remodeling and vessel growth rather than clearance, natural killer cell cytotoxicity against ectopic cells is reduced, and the resulting local milieu tolerates survival rather than opposing it [3,4].

The cumulative effect is a self-reinforcing cycle rather than a single triggering event: adhesion enables invasion, invasion and immune evasion together allow survival long enough for angiogenesis to catch up, and once a lesion has its own vasculature and an immune environment tuned to tolerate it, chronic low-grade inflammation becomes both a consequence of the lesion’s presence and a further driver of its growth. This is the sense in which endometriosis behaves as a systemic dysregulation of the peritoneal microenvironment rather than a disorder localized purely to hormone-responsive ectopic tissue [1].

Retrograde menstruation does not fully account for all disease presentations, including rare cases described in men and endometriosis found at extrapelvic sites unlikely to be reached by tubal reflux. Coelomic metaplasia, in which peritoneal mesothelium transforms into endometrial-like tissue in situ, and Müllerian remnant theories accounting for some congenital or early-onset presentations, remain plausible complementary explanations for a subset of cases [19]. Current opinion increasingly treats these mechanisms as coexisting routes to a common downstream biology rather than as competing, mutually exclusive theories [1,2].

A further complication for a purely mechanical account of retrograde seeding is that retrograde menstruation occurs in most menstruating women, yet only a minority go on to develop endometriosis, which places the explanatory weight on the post-seeding survival and immune-evasion steps described above rather than on the seeding event itself [1]. Two further histogenetic theories belong alongside coelomic metaplasia and the Müllerian remnant explanation. The lymphatic and vascular metastasis theory, proposed by Halban and later revisited by Sampson himself, holds that endometrial cells reach distant and extrapelvic sites, including lymph nodes and lung, through lymphatic or hematogenous spread rather than direct peritoneal reflux, and accommodates disease found outside the pelvis better than retrograde menstruation alone can [19]. A revised stem cell theory proposes instead that endometrial or bone-marrow-derived stem and progenitor cells, rather than fully differentiated endometrial fragments, seed ectopic sites and only subsequently differentiate into glandular and stromal tissue, a model compatible with both retrograde and vasculolymphatic routes and with lesion recurrence after apparently complete surgical excision [20]. These theories are not mutually exclusive, and the most defensible current position treats them as overlapping, possibly co-occurring routes to a common downstream biology rather than as competing accounts of a single uniform process.

## 3. Hormonal Dysregulation and Progesterone Resistance

Estrogen dependence is the oldest and most therapeutically exploited feature of endometriosis, but the way ectopic tissue handles estrogen differs from the way eutopic endometrium does, and this difference is central to why lesions persist despite treatments designed to lower systemic estrogen levels. Aromatase, the enzyme that converts androgens to estrogens, is aberrantly expressed in ectopic endometrial tissue but is essentially absent from normal endometrium. This gives ectopic lesions the capacity to synthesize estrogen locally, largely independent of ovarian production, which helps explain why suppressing ovarian estrogen output does not always translate into lesion regression or symptom control. Compounding this, the enzyme responsible for inactivating estradiol to the weaker estrone, 17β-hydroxysteroid dehydrogenase type 2, is underexpressed in ectopic tissue, so local estrogen is both produced in excess and cleared more slowly than it should be [2].

Receptor expression compounds the exposure problem. Ectopic lesions tend to show a shift in the balance of estrogen receptor isoforms, with relatively increased expression of estrogen receptor beta (ERβ) compared with eutopic endometrium. This shift has functional consequences beyond simple estrogen sensitivity: ERβ signaling has been linked to suppressed apoptosis in ectopic stromal cells and to amplified inflammatory cytokine production, meaning the receptor imbalance contributes directly to lesion survival and to the inflammatory phenotype discussed in the following section, not merely to proliferation [2].

Progesterone signaling shows the mirror-image problem. Progesterone would normally oppose estrogen-driven proliferation and inflammation, and progestin-based therapy relies on this antagonism. In endometriotic tissue, however, cells display a blunted response to progesterone generally described as progesterone resistance. Part of the explanation lies in the progesterone receptor itself: the ratio of the two principal isoforms, progesterone receptor A (PR-A) and progesterone receptor B (PR-B), shifts toward PR-A dominance in ectopic tissue, and since PR-B carries most of progesterone’s transcriptional activity in the endometrium, its relative loss blunts hormone responses even when circulating progesterone levels are adequate [21,22]. This is compounded by disrupted expression of progesterone-responsive genes further downstream in the signaling cascade, so that even the residual receptor activity that remains often fails to produce the expected anti-proliferative and anti-inflammatory transcriptional program [23].

The combined effect, local estrogen excess paired with a blunted progesterone response, creates a hormonal environment that continually favors proliferation and inflammation over quiescence, regardless of circulating hormone levels. This is also the most direct molecular explanation for a pattern familiar from clinical practice: a meaningful proportion of patients do not respond adequately to progestin-based therapy, and hormonal suppression that works well initially can lose effectiveness as lesions persist. If progesterone resistance is intrinsic to ectopic tissue rather than a dosing problem, hormonal therapy alone treats only one axis of a disease that, as later sections describe, is sustained by inflammatory, vascular, and metabolic programs that do not depend on hormone exposure to remain active once established [21,22].

## 4. Inflammatory and Immune Signaling

If hormonal dysregulation explains why ectopic tissue is exposed to a growth-favorable environment, inflammatory and immune signaling explain why that tissue survives contact with a peritoneal cavity that should, in principle, be capable of clearing it. The peritoneal fluid of women with endometriosis is reliably different in composition from that of unaffected women: it contains an expanded population of activated macrophages, altered lymphocyte subsets, and higher concentrations of an overlapping set of pro-inflammatory cytokines and chemokines that recur across multiple independent lines of investigation [3,4].

Macrophages sit at the center of this altered environment. Rather than performing their expected surveillance and clearance function, peritoneal macrophages in endometriosis are recruited in larger numbers and adopt a phenotype weighted toward tissue repair and angiogenic support rather than phagocytic clearance [24]. This has a double cost: ectopic fragments that should be removed are instead tolerated, and the macrophages themselves become active contributors to lesion growth through secretion of angiogenic and growth-promoting factors into the local environment. Natural killer cell function follows a related pattern, with reduced cytotoxic activity against endometrial cells reported in the peritoneal environment of affected women [25], further weakening one of the few remaining mechanisms that might otherwise limit ectopic tissue survival. T lymphocyte populations shift as well, with evidence for an imbalance favoring pro-inflammatory Th17 responses over regulatory T cell activity, tilting local immune tone further toward chronic inflammation rather than tolerance or clearance [4,26,27].

The macrophage shift described above has an identifiable molecular basis: local IL-4, IL-13, and macrophage colony-stimulating factor, amplified by estrogen signaling through ERβ, engage the IL-4 receptor alpha chain to activate JAK kinases and STAT6, and STAT6 in turn drives the transcriptional program (CD163, arginase, IL-10) responsible for the repair- and angiogenesis-biased phenotype rather than the pathogen-clearing program macrophages default to elsewhere in the body [3]. The Th17/Treg balance follows a parallel logic: TGF-β acting together with IL-6, IL-21, and IL-23 favors RORγt-driven Th17 differentiation over Foxp3-driven regulatory T cell differentiation, and interleukin-27 further redirects a subset of Th17 cells toward an IL-10-producing, immunosuppressive phenotype through a c-Maf/RORγt/Blimp-1 complex, a conversion that paradoxically supports lesion tolerance rather than clearance as disease advances [28].

The cytokine and chemokine profile generated by this altered cellular environment reads as a catalogue of chronic inflammation: TNF-α, IL-6, and IL-1β are consistently elevated in peritoneal fluid and are each capable of promoting endometrial cell proliferation, adhesion, and further cytokine release, creating feed-forward loops rather than a single transient inflammatory burst. Chemokines, particularly MCP-1, help sustain recruitment of additional macrophages into the peritoneal cavity, perpetuating the cycle rather than allowing it to resolve. Cyclooxygenase-2 and its downstream product prostaglandin E2 add a further layer, since PGE2 contributes directly to pain signaling and also stimulates local aromatase activity, linking the inflammatory and hormonal axes described in the previous section into a single reinforcing circuit [2,29].

NF-κB and JAK/STAT signaling integrate most of this activity into two intracellular hubs; their full molecular cascades are detailed in Section 8.3 and Section 8.4 rather than repeated here. In brief, NF-κB functions as the switch that keeps the cytokine cascade described above running once triggered, while JAK/STAT propagates IL-6 signaling and helps ectopic cells resist apoptotic cues that would otherwise limit their persistence [29,30].

Taken together, these signals show that inflammation is not an incidental consequence of ectopic tissue being present, the way a splinter might provoke local irritation, but an active participant in the disease process. Chronic inflammation supports lesion survival, drives the angiogenic and neurogenic programs discussed next, and generates much of the pain symptomatology that brings patients to clinical attention. Any therapeutic strategy that addresses hormonal signaling without also addressing this inflammatory circuit is, by this logic, treating only part of what keeps the disease active [31].

## 5. Angiogenesis, Lymphangiogenesis, and Neurogenesis

For an ectopic lesion to grow beyond what passive diffusion of oxygen and nutrients can support, it needs its own vascular supply, and endometriotic tissue is unusually effective at building one. Vascular endothelial growth factor, principally VEGF-A, is markedly upregulated in both eutopic and ectopic endometrium in women with the disease, driven by the combined effects of local hypoxia and the inflammatory cytokines described in the previous section. Hypoxia-inducible factor 1-alpha, stabilized under the low-oxygen conditions that shed and displaced endometrial fragments initially encounter, functions as a master regulator here, upregulating VEGF transcription and linking the metabolic stress of the ectopic environment directly to the vascular program that allows the lesion to overcome it [5,18].

This picture is incomplete if it stops at HIF-1α. HIF-1α and HIF-2α share some angiogenic targets, VEGF-A among them, but operate on different timescales: HIF-1α dominates the acute response to oxygen deprivation, while HIF-2α becomes the principal regulator once hypoxia persists beyond roughly a day, a handoff described as the HIF switch [13]. Endometriotic lesions experience recurrent, cyclical hypoxia rather than a single acute insult, so HIF-2α is plausibly at least as relevant to the later, sustained phase of lesion vascularization as HIF-1α, yet it has received little dedicated attention in endometriosis. The relationship between hypoxia and inflammation is also bidirectional rather than one-way: HIF stabilization upregulates proinflammatory cytokines including IL-1β and TNF-α, and these cytokines, largely through NF-κB signaling, in turn stabilize and activate HIF, producing a loop in which hypoxia and inflammation continuously reinforce each other rather than hypoxia simply triggering a single angiogenic response [32].

TGF-β adds a further layer to this vascular and structural remodeling. Beyond its immunomodulatory role in shaping the tolerant peritoneal environment discussed above, TGF-β promotes fibrogenesis, contributing to the fibrotic component that characterizes many endometriotic lesions, particularly deep infiltrating disease, and to the tissue remodeling that accompanies lesion expansion. Matrix metalloproteinases, already noted for their role in initial peritoneal invasion, continue to operate throughout lesion growth, degrading extracellular matrix components to make room for expanding vasculature and infiltrating stromal tissue alike [33].

Neurogenesis is the aspect of this vascular and structural remodeling most directly relevant to the symptom that drives most patients to seek care: chronic pelvic pain. Endometriotic lesions are innervated by both sensory and autonomic nerve fibers at densities well above those found in normal peritoneum, and this new nerve growth appears tightly coupled to the angiogenic program, closely enough that the process is often described jointly as neuroangiogenesis rather than treated as two separate phenomena. Nerve growth factor and related neurotrophins, produced locally within lesions, support this innervation and appear to sensitize the nerve fibers that grow in, contributing to a state of peripheral and, over time, central sensitization that can decouple the intensity of a patient’s pain from the visible extent of disease found at surgery [34,35].

This decoupling has real clinical consequences. Lesion burden at laparoscopy correlates poorly with reported pain severity, a familiar and often frustrating pattern in practice, and the neuroangiogenesis model offers a plausible molecular explanation: pain in this disease is generated locally by a purpose-built vascular and neural infrastructure rather than simply signaling the presence of tissue, and once central sensitization has developed, pain can persist or worsen even after surgical excision has removed the lesions that originally triggered it. This has practical implications for how success is measured in both clinical practice and trial design, since resolution of visible disease and resolution of pain are not interchangeable outcomes [35].

## 6. Epigenetic Regulation

Much of the biology described so far, hormonal receptor imbalance, chronic inflammatory signaling, and aberrant vascular and neural growth, raises an obvious question: what locks these changes in place well enough that they persist across the lifespan of a lesion and, in many cases, resist attempts to reverse them hormonally? Epigenetic regulation, encompassing DNA methylation, histone modification, and a growing catalogue of non-coding RNAs, offers much of the answer and has become a particularly active area of endometriosis research over the past decade [6].

DNA methylation changes affect several of the genes already discussed in earlier sections, which is itself informative: rather than introducing new mechanisms, epigenetic dysregulation appears largely to entrench mechanisms already identified elsewhere in this review. HOXA10, a gene central to normal endometrial receptivity and differentiation, is hypermethylated in the eutopic endometrium of women with the disease, plausibly contributing to the endometrial receptivity defects associated with endometriosis-related infertility [36]. Estrogen and progesterone receptor genes show altered promoter methylation as well, in patterns that favor the ERβ-dominant, PR-B-deficient receptor profile addressed in Section 3, suggesting that epigenetic marks may be one mechanistic layer underlying that receptor imbalance rather than a separate, unrelated phenomenon [6].

This repressive effect is mostly indirect: methyl-CpG-binding proteins such as MeCP2 recognize methylated cytosines and recruit histone deacetylase-containing corepressor complexes, converting a DNA-level mark into a condensed, transcriptionally silent chromatin state rather than blocking transcription factor binding on its own [16].

Histone modifications add a further regulatory layer. Histone deacetylases (HDACs) are overexpressed in ectopic endometrial tissue [37], and their activity generally favors a more condensed, transcriptionally repressive chromatin state at specific loci, including genes involved in cell cycle regulation and apoptosis, favoring cell survival over the controlled turnover expected in normal endometrium [6].

More generally, acetylation of histone lysine residues neutralizes their positive charge and loosens histone–DNA contact, opening chromatin to transcription factor binding, while deacetylation and specific methylation marks restore a condensed state that excludes the transcriptional machinery; it is the balance of these opposing marks at a given locus, rather than any single modification considered alone, that determines whether that locus stays accessible [17].

Non-coding RNAs have generated the most clinical interest, largely because they are detectable in peripheral blood and therefore carry biomarker potential alongside their mechanistic role. Several microRNAs recur across independent studies of endometriotic tissue and peritoneal fluid: members of the let-7 family and miR-29c, generally downregulated in disease, normally restrain fibrotic and epithelial–mesenchymal transition (EMT) programs, so their loss is consistent with the enhanced invasive and fibrotic phenotype of ectopic lesions; the miR-200 family, similarly implicated in epithelial–mesenchymal balance, shows altered expression patterns that parallel the invasive behavior of ectopic stromal cells [38,39]. Long non-coding RNAs, including MALAT1 and HOTAIR, are upregulated in ectopic tissue and appear to support proliferation and invasion through interactions with chromatin-modifying complexes and by acting as molecular scaffolds influencing the transcriptional programs described above. Circular RNAs represent the newest addition to this catalogue, with an expanding body of work implicating specific circRNA species in the regulation of proliferation, invasion, and inflammatory signaling in ectopic endometrial cells, though this field remains earlier in its development than the microRNA and lncRNA literature [40].

The restraint these microRNAs impose has identifiable molecular targets. Let-7 represses RAS family GTPases and the chromatin regulator HMGA2 [14]. In endometriotic cells specifically, miR-29c-3p downregulates the cell-cycle regulators CDK6 and BCCIP along with the transcription factors TCF7L1 and TCF7L2, and its loss is associated with increased invasion and proliferation of endometriotic cells [41]. The miR-200 family represses the transcriptional repressors ZEB1 and ZEB2, whose de-repression upon miR-200 loss drives the epithelial-to-mesenchymal shift underlying ectopic cell invasiveness [15]. Small interfering RNAs and PIWI-interacting RNAs (piRNAs) complete the catalogue of small regulatory RNA classes but remain largely uncharacterized in endometriosis specifically; piRNAs, better studied in germline and malignant tissue, silence transposable elements and target genes through PIWI-protein-guided DNA methylation, a mechanism plausible in ectopic endometrial tissue given the broader epigenetic dysregulation described above, though dedicated endometriosis studies of piRNA expression are still lacking.

The practical significance of this layer of biology is twofold. First, epigenetic marks offer a plausible explanation for treatment resistance: if a hormonal or inflammatory pathway has been epigenetically reinforced, removing the original hormonal trigger alone may not be sufficient to reverse the downstream chromatin state, which would explain why some lesions persist or recur even after seemingly adequate hormonal suppression. Second, because several of the microRNAs and other non-coding RNAs described here are stable in peripheral blood, they represent an especially promising source of the non-invasive biomarkers taken up in Section 10, potentially shortening the diagnostic delay that currently affects most patients [6,42].

## 7. Metabolic Reprogramming and Hypoxia

Ectopic endometrial tissue does not merely tolerate the hostile, low-oxygen environment of the peritoneal cavity into which it has been displaced; it actively reprograms its metabolism to exploit that environment, in a pattern with clear parallels to tumor metabolism even though endometriosis remains, in every meaningful clinical sense, a benign disease. This parallel has become an unusually productive framing in recent endometriosis research, since it allows investigators to borrow both concepts and candidate drug targets from a much larger body of cancer metabolism literature [7].

This borrowed vocabulary, Warburg-like metabolism, invasion, and angiogenesis, describes convergent biological behavior rather than a shared disease category: endometriosis remains a benign condition without the genomic instability, metastatic capacity, or mortality risk that define malignancy, and the analogy is used throughout this review as a source of mechanistic hypotheses and repurposable drug targets rather than as a claim of biological equivalence with cancer.

Hypoxia is the starting point. Shed endometrial fragments are, by definition, displaced from their normal blood supply, and even after a lesion has established some vascularization through the angiogenic program outlined in Section 5, cyclic episodes of hypoxia and reoxygenation appear to persist within lesions, generating recurrent oxidative stress. HIF-1α, stabilized under these low-oxygen conditions, does more than drive the VEGF-dependent angiogenic response already discussed: it also reprograms cellular metabolism directly, upregulating glycolytic enzymes and the glucose transporter GLUT1 so that ectopic cells shift toward glycolysis for energy production even when oxygen is available, a pattern that mirrors the Warburg effect long described in tumor cells [7]. This glycolytic shift is metabolically less efficient per glucose molecule than oxidative phosphorylation, but it offers a decisive advantage in a hypoxic, poorly perfused microenvironment, since it does not depend on oxygen availability and can support rapid proliferation using a substrate, glucose, generally in adequate local supply.

The recurrent hypoxia–reoxygenation cycling associated with this environment also generates reactive oxygen species in excess of what normal cellular antioxidant defenses can neutralize, producing a state of chronic oxidative stress within lesions. This oxidative burden appears to further damage mitochondrial function, compounding the shift away from oxidative metabolism and contributing to a self-reinforcing metabolic phenotype: damaged mitochondria favor glycolysis, and sustained glycolytic dependence in turn reduces the pressure on cells to maintain full mitochondrial function [7].

The hypoxia sensitivity considered so far is local, arising from a lesion’s own vascular insufficiency, but organisms also differ systemically in how well they tolerate oxygen deprivation, and this baseline tolerance is not evenly distributed across a population. In experimental work comparing hypoxia-tolerant and hypoxia-susceptible animals, susceptible individuals show a higher constitutive proinflammatory potential, with elevated HIF-1α, HIF-2α, and HIF-3α alongside NF-κB in peripheral blood leukocytes, and this susceptible phenotype tracks with more severe systemic inflammatory responses and faster tumor progression once disease is present [43,44,45]. Sex differences add a further layer: in these models, females are consistently more tolerant of hypoxia than males [43]. Whether a woman’s constitutional, systemic hypoxia tolerance, as distinct from the local hypoxia her ectopic lesions experience, contributes to her likelihood of developing endometriosis or to disease severity is a question endometriosis-specific research has not yet addressed, but the underlying principle, that systemic hypoxia tolerance shapes susceptibility to inflammatory and proliferative disease elsewhere in the body, is established enough to warrant investigation here [45].

The PI3K/AKT/mTOR axis functions as the principal integrator linking this metabolic reprogramming to the proliferative and survival phenotypes described throughout this review. This pathway responds to the growth factor and cytokine signaling already discussed in the inflammatory and angiogenic sections, and its activation supports glycolytic gene expression, protein synthesis needed for continued proliferation, and resistance to the apoptotic signals that would normally eliminate cells surviving in such an inhospitable environment. PI3K/AKT/mTOR therefore sits at the intersection of nearly every mechanism covered elsewhere in this review, metabolic, proliferative, and survival-related alike, which is a large part of why it recurs prominently in the discussion of therapeutic targets that follows; the pathway’s specific molecular components are detailed in Section 8.2 [7,29]. The interconnected relationships among these reinforcing processes are summarized in Figure 1.

## 8. Key Molecular Pathways

The mechanisms described in the preceding sections converge on a relatively compact set of intracellular signaling pathways, several of which have already appeared repeatedly under different headings. Rather than treat each pathway as belonging exclusively to one biological process, hormonal, inflammatory, vascular, or metabolic, it is more accurate to describe them as shared infrastructure recruited by multiple upstream signals and directed toward a common set of downstream outcomes: proliferation, survival, invasion, and immune evasion. This section draws these pathways together explicitly, both to consolidate the mechanistic picture built so far and to set up the therapeutic discussion that follows, since a pathway’s value as a drug target depends heavily on how many of the disease’s downstream manifestations it actually controls [29].

### 8.1. MAPK/ERK Signaling

The MAPK/ERK cascade, activated downstream of growth factor receptors and by several of the inflammatory cytokines already discussed, principally supports proliferation, migration, and survival in ectopic endometrial stromal and epithelial cells. Estrogen signaling through membrane-associated receptor pools can activate this cascade rapidly and independently of classical genomic estrogen receptor action, providing one mechanistic link between the hormonal environment introduced in Section 3 and the proliferative behavior of ectopic tissue. ERK activation also appears to potentiate the invasive phenotype of ectopic cells by upregulating matrix metalloproteinase expression, connecting this pathway directly to the invasion step covered in Section 2 [29].

In molecular terms, growth factor receptors and membrane estrogen receptor signaling activate the Ras-Raf-MEK1/2-ERK1/2 cascade; activated ERK1/2 moves into the nucleus and phosphorylates transcription factors including Elk-1 and components of AP-1, driving the proliferative and MMP-dependent transcriptional program described above.

### 8.2. PI3K/AKT/mTOR Pathway

Building on Section 7, PI3K/AKT/mTOR signaling links growth factor and cytokine input to cell survival, protein synthesis, and the glycolytic metabolic program that allows ectopic cells to tolerate a hypoxic microenvironment. Beyond its direct proliferative and metabolic roles, this pathway actively suppresses apoptosis, in part through downstream inhibition of pro-apoptotic BCL-2 family members, helping explain why ectopic tissue survives outside its normal anatomical context rather than undergoing the programmed cell death expected of displaced endometrial fragments [7,29].

At the molecular level, PI3K phosphorylates membrane phosphoinositides to recruit and activate AKT, which inhibits the TSC1/TSC2 complex and thereby relieves suppression of Rheb, activating mTORC1; mTORC1 phosphorylates S6 kinase and 4E-BP1 to drive protein synthesis and the glycolytic gene program, while AKT separately phosphorylates and inactivates the pro-apoptotic proteins BAD and FOXO3a, the specific point at which this pathway blocks apoptosis.

### 8.3. NF-κB Pathway

NF-κB functions as the central transcriptional hub of the inflammatory phenotype examined in Section 4, integrating input from TNF-α, IL-1β, and other cytokine signals and translating it into sustained expression of additional pro-inflammatory genes, adhesion molecules, and factors supporting angiogenesis and invasion. Because so many upstream signals converge on NF-κB and because its output touches nearly every downstream process described in this review, it is frequently identified as one of the most attractive, and most difficult, therapeutic targets in endometriosis: attractive because of its central position, difficult because broad NF-κB inhibition risks compromising immune function needed elsewhere in the body [29,30].

Mechanistically, upstream cytokine signaling activates the IκB kinase (IKK) complex, which phosphorylates the inhibitory protein IκBα and marks it for proteasomal degradation; this releases the p65/p50 NF-κB dimer to enter the nucleus and drive transcription of the cytokine, adhesion molecule, and angiogenic genes described in Section 4, an activity reinforced by the hypoxia–inflammation feedback loop discussed in Section 5 [32].

### 8.4. JAK/STAT Signaling

JAK/STAT signaling operates largely downstream of IL-6 and related cytokines, sustaining the chronic inflammatory state set out in Section 4 and contributing an additional survival signal that complements PI3K/AKT/mTOR activity. Its role appears to be less about initiating disease processes and more about maintaining them once established, which makes it a plausible target for preventing disease persistence and recurrence rather than addressing initial lesion establishment [46].

IL-6 binding to its receptor complex activates JAK1 and JAK2, which phosphorylate STAT3; phosphorylated STAT3 dimerizes and enters the nucleus to drive a survival and proliferative program that overlaps substantially with the output of PI3K/AKT/mTOR, one reason the two pathways are often considered jointly as a combined therapeutic target.

### 8.5. TGF-β and EMT-Related Signaling

TGF-β signaling connects the immunomodulatory and fibrogenic processes sketched in Section 5, promoting an epithelial-to-mesenchymal transition-like phenotype shift in ectopic endometrial epithelial cells that increases their migratory and invasive capacity. This EMT-like transition compounds the fibrotic component already noted in deep infiltrating disease (Section 5), linking this pathway to a clinically challenging subtype in which fibrosis, rather than active proliferation, often drives symptoms and surgical difficulty [33].

TGF-β signals through TGFBR1/TGFBR2 receptor complexes to phosphorylate SMAD2 and SMAD3, which partner with SMAD4 to regulate transcription of SNAI1, SNAI2, and ZEB1/ZEB2, the same E-cadherin repressors discussed in Section 6 as targets of microRNA regulation, directly connecting this pathway’s EMT-driving output to the epigenetic biology described there [15].

### 8.6. Wnt/β-Catenin, Rho/ROCK, and Hedgehog Signaling

A further cluster of developmental signaling pathways, less extensively characterized than those above but increasingly implicated in endometriosis, contributes to the migratory and adhesive behavior of ectopic cells and to remodeling of the surrounding microenvironment. Wnt/β-catenin signaling, aberrantly activated in ectopic tissue, supports both proliferation and the acquisition of a more mesenchymal, invasive cell phenotype. Rho/ROCK signaling regulates cytoskeletal dynamics underlying cell migration and appears to support the invasive behavior of ectopic stromal cells at the tissue level. Hedgehog pathway components, more familiar from developmental biology and from a range of malignancies, have been detected at elevated levels in ectopic lesions, though their precise functional contribution remains less well defined than that of the pathways discussed above. Collectively, these pathways are best understood as auxiliary systems that reinforce the adhesive, migratory, and remodeling behaviors already established by the more central pathways, rather than as independent disease drivers [29,47].

Wnt ligands bind Frizzled and LRP5/6 co-receptors to inhibit the destruction complex that would otherwise mark β-catenin for degradation, allowing it to accumulate and enter the nucleus, where it partners with TCF/LEF transcription factors, including the TCF7L1 and TCF7L2 targets of miR-29c-3p noted in Section 6, to drive proliferative and mesenchymal gene programs. Rho/ROCK signaling acts further downstream, with active RhoA recruiting ROCK1/ROCK2 to phosphorylate and inhibit the actin-severing protein cofilin, stabilizing a cytoskeletal configuration that favors migration [41].

Of the pathways surveyed in this section, NF-κB and PI3K/AKT/mTOR stand out as the most promising therapeutic targets, not because their individual biology is better understood than that of the others, but because each sits at a convergence point where multiple upstream signals, hormonal, inflammatory, and metabolic, meet and are translated into the downstream behaviors that define the disease. Targeting either pathway offers the theoretical prospect of addressing several disease mechanisms simultaneously rather than one in isolation. JAK/STAT and TGF-β signaling represent a second tier of interest, valuable less for initiating new therapeutic strategies than for combination approaches aimed at disease maintenance and fibrotic subtypes respectively. MAPK/ERK and the developmental pathways covered in Section 8.6 remain scientifically important for understanding disease biology but currently offer less differentiated therapeutic opportunity, in part because their broad roles in normal tissue homeostasis raise the same selectivity concerns that complicate NF-κB-directed therapy, without the compensating advantage of comparably central disease relevance [29,46]. These pathways, their principal biological functions, and candidate therapeutic targets are summarized in Figure 2 and Table 1.

To make the strength of evidence underlying each pathway explicit rather than embedding it in free text, this review classifies supporting evidence into four tiers: Tier I, human interventional or clinical trial evidence specific to endometriosis; Tier II, human tissue or correlative studies (e.g., immunohistochemistry, expression profiling) supported by convergent preclinical data; Tier III, preclinical evidence only, from in vitro and/or animal models, with no endometriosis-specific human data; and Tier IV, exploratory human tissue findings alone (e.g., elevated expression or localization), without functional or interventional evidence. This classification, summarized in the final column of Table 1 and applied consistently to the therapeutic strategies in Table 2, is intended to separate experimentally validated mechanisms from mechanistically plausible but clinically untested hypotheses; a Tier III or Tier IV classification should not be read as evidence of clinical applicability.

## 9. Therapeutic Targets and Emerging Strategies

Translating the mechanistic picture assembled above into therapy requires distinguishing between approaches already in clinical use, those in active clinical development, and those that remain largely preclinical. The subsections below are organized by mechanism rather than by developmental stage, but the distinction matters throughout: hormonal therapies benefit from decades of clinical experience, while several of the mechanistically most attractive strategies, particularly those targeting epigenetic or metabolic pathways, remain early-stage work whose eventual clinical value is still uncertain. Each strategy discussed below is labeled in Table 2 by both its developmental stage and its evidence tier, using the classification introduced in Section 8 (Table 1); Tier III and Tier IV strategies in particular should be read as mechanistically motivated hypotheses awaiting endometriosis-specific human evidence rather than as approaches with established or even preliminary clinical applicability in this disease.

### 9.1. Hormonal Therapies

Progestins remain first-line medical therapy, exploiting the antiproliferative and anti-inflammatory effects of progesterone signaling treated in Section 3, though their efficacy is inherently limited in patients whose ectopic tissue shows pronounced progesterone resistance. GnRH agonists and, more recently, oral GnRH antagonists such as elagolix and relugolix suppress ovarian estrogen production and have become an increasingly favored option because they avoid the initial flare associated with agonists and can be combined with low-dose add-back hormone therapy to limit hypoestrogenic side effects, though they do little to address the locally produced estrogen generated by aberrant aromatase activity within lesions themselves [48]. Aromatase inhibitors target this local synthesis directly and have shown benefit particularly in patients who fail conventional hormonal suppression, though they are generally reserved for refractory cases given their side-effect profile when used alone in premenopausal women [49]. Selective progesterone receptor modulators offer a further mechanistic angle, aiming to engage residual progesterone receptor activity more effectively despite the PR-A/PR-B imbalance discussed above, though their long-term role in endometriosis specifically remains less established than in uterine fibroids, where this drug class is more mature [22].

None of these hormonal approaches modifies the underlying disease process in the sense of reversing established lesions; all suppress the hormonal drive behind proliferation and inflammation for as long as they are taken, which is why symptom relief and lesion regression should not be treated as interchangeable outcomes, and why symptoms characteristically recur once therapy stops.

### 9.2. Anti-Inflammatory Approaches

Direct cytokine blockade has been tested most extensively with anti-TNF-α agents, building on the strong preclinical rationale provided by TNF-α’s role in the inflammatory circuit noted in Section 4; clinical results, however, have been considerably less encouraging than the mechanistic rationale suggested, and this approach has not translated into an established treatment option, illustrating a recurring theme in this field where compelling cell and animal model data does not reliably predict clinical benefit [31]. COX-2 inhibitors address the prostaglandin arm of the inflammatory pathway and provide meaningful symptomatic pain relief for some patients, but cardiovascular safety concerns limit their use to short-term symptom control rather than long-term disease-modifying therapy [2]. NF-κB and JAK/STAT-directed strategies remain largely investigational in this specific disease context, borrowing agents developed for other inflammatory and autoimmune conditions; their central position in the pathway map from Section 8 makes them mechanistically appealing, but achieving adequate local disease suppression without excessive systemic immunosuppression remains an unresolved challenge for this entire drug class [30,46].

As with hormonal suppression, the anti-inflammatory strategies above are aimed principally at symptom control, pain and, to a lesser extent, the inflammatory drive behind lesion persistence, rather than at reversing established fibrotic or invasive change; this distinction is particularly relevant for COX-2 inhibitors, whose benefit is confined to pain during use rather than any lasting effect on disease course.

### 9.3. Anti-Angiogenic and Anti-Neurogenic Strategies

Approaches targeting VEGF signaling, extensively validated in oncology, have an obvious theoretical rationale in a disease so dependent on lesion-specific vascularization, but clinical development specific to endometriosis remains early, constrained partly by legitimate concern about anti-angiogenic effects on normal reproductive tissue in a patient population where fertility preservation often matters a great deal [5,18]. HIF-1α-directed strategies face a similar rationale-versus-translation gap. Approaches aimed at the neuroangiogenic component specifically, including agents targeting nerve growth factor signaling, are of particular interest given the disconnect, raised in Section 5, between visible lesion burden and reported pain severity; a therapy able to interrupt local nerve sensitization independent of lesion size would address a treatment gap that purely anti-proliferative approaches cannot [34].

Anti-neurogenic strategies illustrate this distinction most sharply: interrupting nerve sensitization could relieve pain substantially without shrinking a single lesion, which would count as a major clinical success for the patient while leaving the underlying disease process entirely unmodified, a combination current outcome measures do not always capture cleanly.

### 9.4. Epigenetic Therapies

Histone deacetylase inhibitors, developed originally in oncology, have shown activity against ectopic endometrial cells in preclinical models, consistent with the HDAC overexpression documented in Section 6, though clinical development in endometriosis specifically remains preliminary [6]. MicroRNA-based strategies, either mimics restoring the activity of downregulated species such as let-7 and miR-29c, or antagomirs suppressing overexpressed lncRNAs and their downstream effects, represent a conceptually elegant approach to reversing specific epigenetic lesions rather than suppressing hormone signaling broadly, though delivery of nucleic acid therapeutics specifically to peritoneal lesions remains a substantial practical obstacle before this class of agent could reach clinical use [38,39].

### 9.5. Metabolism-Targeted Interventions

mTOR inhibitors, again drawing on extensive oncology experience, offer a route to addressing the proliferative and glycolytic phenotype detailed in Section 7 directly, and preclinical work supports activity against ectopic lesion growth, though systemic mTOR inhibition carries a side-effect burden that has so far limited enthusiasm for extended use in a benign, chronic condition [7]. Metformin, already widely used and well tolerated in other metabolic contexts, has drawn interest as a repurposed candidate given its effects on cellular energy sensing and mild anti-inflammatory activity, offering a more favorable safety profile for long-term use than more potent oncology-derived agents, even though the evidence base specific to endometriosis remains limited [50]. Antioxidant strategies aimed at the oxidative stress component of the hypoxia–reoxygenation cycle have been explored as adjunctive rather than primary therapy, with modest and inconsistent results to date [51].

### 9.6. Immunomodulatory and Precision Approaches

Rather than targeting a single cytokine or pathway, this category envisions stratifying patients by their dominant inflammatory or immune profile, whether macrophage-driven, Th17-skewed, or NK-cell-deficient, and matching immunomodulatory therapy accordingly. This approach remains more conceptual than clinically available today, but it follows directly from the heterogeneity documented throughout the immune and inflammatory sections of this review. It also depends heavily on the biomarker and precision medicine infrastructure developed in Section 10, since immune-profile-guided therapy is only actionable once immune profiling itself becomes a practical, validated clinical tool rather than a research exercise [4,26]. Table 2 groups the therapeutic strategies discussed in this section by developmental stage, distinguishing established treatments from investigational, repurposed, and preclinical approaches rather than presenting them within a single undifferentiated framework.

## 10. Biomarkers and Precision Medicine

Diagnostic delay remains a persistent problem in endometriosis care. Laparoscopic visualization, ideally with histological confirmation, was long treated as the diagnostic reference standard, but current ESHRE guidance no longer frames it this way: laparoscopy is now recommended only when imaging is negative or empirical hormonal treatment has failed, with transvaginal ultrasound and MRI serving as first-line diagnostic tools in typical presentations [52]. A validated non-invasive biomarker, or biomarker panel, would nonetheless address the delay that persists even under this revised pathway, and would open the door to something the disease currently lacks almost entirely: an objective, biologically grounded way to match individual patients to the treatment most likely to work for their particular phenotype, rather than proceeding through a largely trial-and-error sequence of hormonal options [53]. The candidates discussed below serve, at least potentially, several distinct clinical purposes, diagnosis, disease or lesion stratification, prognosis, prediction of treatment response, and longitudinal monitoring, that are frequently conflated in the literature despite requiring different validation standards.

For diagnosis specifically, CA-125, the longest-studied candidate, is elevated in a meaningful proportion of patients with more advanced disease but lacks the sensitivity and specificity needed for reliable standalone use, particularly in earlier-stage disease, and its levels are influenced by numerous other gynecological conditions. Panels combining multiple inflammatory cytokines, drawing on the altered cytokine profile outlined in Section 4, have shown somewhat better discriminatory performance than single markers in various cohorts, and circulating microRNA signatures, building on the epigenetic biology introduced in Section 6, represent an actively investigated newer diagnostic approach; current ESHRE guidance nonetheless recommends against relying on any circulating biomarker for diagnosis outside a research setting, reflecting how far this particular application remains from clinical readiness [52].

Stratification, matching a patient’s dominant molecular phenotype, inflammatory, angiogenic, epigenetic, or metabolic, to a specific therapeutic approach, is conceptually distinct from diagnosis and depends less on distinguishing affected from unaffected women than on distinguishing among affected women. Peritoneal fluid profiling and tissue or lesion multi-omics profiling, integrating genomic, transcriptomic, epigenomic, and metabolomic data, both serve this stratification purpose more directly than a diagnostic one, since obtaining either sample generally requires the surgical access that the biomarker would ideally help patients avoid for diagnosis; their more realistic near-term role is stratifying and characterizing disease in patients already undergoing surgery, and increasingly defining molecular subtypes that may correspond to differences in clinical behavior, treatment response, or prognosis [4,54,55].

For prognosis, specifically predicting recurrence after surgery, NF-κB offers a more explicit mechanistic rationale than it is usually given credit for: because it functions as the central transcriptional hub described in Section 4 and Section 8, its activation state, measured for instance as nuclear p65 expression in peripheral blood mononuclear cells, has already shown associations with disease recurrence in preliminary cohorts, though it has not been developed into a standardized prognostic assay [56].

Prediction of treatment response, identifying in advance which patients will respond to a given hormonal, anti-inflammatory, or targeted therapy, remains the least developed of these applications. The mechanistic rationale for a microRNA-based approach is at least specifiable: because let-7, miR-29c, and the miR-200 family act on identifiable targets, RAS, HMGA2, CDK6, TCF7L1/TCF7L2, and ZEB1/ZEB2, that govern the proliferative and invasive behavior described in Section 6 and Section 8, a panel built around these microRNAs measures a defined mechanistic axis rather than an arbitrary set of disease-associated markers [14,15,41]. No prospective study, however, has yet tested whether baseline expression of these or other candidates predicts response to a specific therapy, so this particular application remains hypothetical rather than validated.

Longitudinal monitoring, tracking disease activity or treatment response over time rather than making a one-time diagnostic or prognostic call, could in principle draw on any of the candidates above, but current ESHRE guidance gives this application only a weak recommendation, reflecting the absence of a properly established long-term evidence base [52].

Across all five applications, the same set of validation problems recurs. Cross-study reproducibility for circulating microRNA panels has been modest, and a systematic review attributed this largely to a combination of genuine biological variability and inconsistent pre-analytical and analytical protocols across centers, including differences in sample handling, storage, and normalization method [42]. Menstrual-cycle phase affects circulating levels of some candidate markers and is not consistently controlled for across studies; cohort composition, particularly the mix of lesion subtypes, disease stage, and control-group definition, varies enough between studies that a panel validated in one population may not generalize to another; and few candidate panels have been tested in the large, prospectively recruited, externally validated cohorts that would be needed to establish real clinical utility rather than promising discriminatory statistics from a single center’s samples. Taken together, these problems explain why none of the circulating biomarkers discussed above has moved from research use into routine clinical implementation: even the most extensively studied candidate, CA-125, is limited by sensitivity and specificity that fall well short of what a standalone diagnostic requires, and the newer candidates, cytokine panels and circulating microRNAs, have not yet been tested in the large, prospectively recruited, externally validated cohorts that regulatory-grade clinical adoption would require. Almost all validation reported to date is internal to the discovering center or study rather than externally replicated in an independent population, which is precisely the gap current ESHRE guidance reflects in declining to recommend any circulating biomarker for diagnostic use outside a research setting [42,52].

The broader ambition uniting this section is a move away from treating endometriosis as a single disease with a uniform hormonal treatment algorithm, and toward a model in which molecular phenotype determines treatment selection much as molecular subtyping increasingly guides therapy in oncology. This ambition is realistic in principle given the mechanistic heterogeneity documented throughout this review, but for every application described above, it remains substantially ahead of the validated biomarker infrastructure needed to put it into everyday practice [26,54]. Table 3 summarizes the candidate biomarkers discussed above, organized by their most realistic clinical application rather than by sample source alone.

## 11. Translational Challenges

The gap between the mechanistic sophistication of current endometriosis research and the relatively modest expansion of the clinical treatment options actually available to patients over the same period deserves direct acknowledgment, since a review of promising pathways and candidate therapies that omits this gap risks reading as a list of undelivered promises rather than a genuinely balanced scientific account [31].

Animal models bear a substantial share of the translational difficulty. Rodents, the most widely used experimental species, do not menstruate, so endometriosis must be induced artificially, typically by autologous or syngeneic transplantation of endometrial tissue into the peritoneal cavity, a method that reproduces some but not all features of spontaneous human disease and that necessarily bypasses the retrograde menstruation step believed central to natural disease initiation [57]. Beyond bypassing retrograde menstruation, induced rodent models typically run over weeks rather than the years to decades over which human disease develops and is treated, which limits their capacity to capture the chronic epigenetic entrenchment described in Section 6 or the cumulative fibrotic remodeling characteristic of deep infiltrating disease; most standard protocols also do not reproduce the native menstrual cyclicity that shapes hormonal and immune signaling in women, so estrogen- and progesterone-pathway findings from these models warrant particular caution before extrapolation to human treatment timelines. Non-human primates, principally baboons and macaques, menstruate and can develop endometriosis spontaneously or following induced retrograde seeding, offering a biologically closer model, but the cost, long experimental timelines, and ethical considerations associated with primate research sharply limit how much of the mechanistic work described in this review has actually been validated in this more representative system. The practical consequence is that a considerable share of the pathway biology discussed above rests on rodent transplantation models, cell culture systems, or human tissue studied outside its native context, each of which captures only part of the disease as it actually behaves in patients [57].

This modeling gap feeds directly into a broader preclinical-to-clinical disconnect that recurs throughout Section 9: anti-TNF therapy, to take one clear example already discussed, performed encouragingly in preclinical models built on the inflammatory mechanisms covered in Section 4, yet failed to demonstrate comparable benefit in clinical trials. This pattern is not unique to endometriosis, but the disease’s reliance on models that imperfectly recapitulate human pathophysiology plausibly makes the field more vulnerable to this kind of disappointment than fields with more representative model systems available [31].

Lesion heterogeneity compounds the problem from a different direction. Peritoneal superficial lesions, ovarian endometriomas, and deep infiltrating endometriosis are increasingly understood to differ meaningfully in their molecular profile and dominant pathophysiology; TGF-β-driven fibrosis appears especially prominent in deep infiltrating disease, for instance, rather than existing merely as different anatomical presentations of one uniform biological process [33,58]. Clinical trials that enroll patients across this full anatomical spectrum without stratifying by lesion subtype risk diluting a genuine treatment effect present in one subtype within a broader, more heterogeneous, and therefore less responsive trial population, one plausible contributor to the mismatch between mechanistic promise and trial outcomes described above.

Table 4 makes this heterogeneity explicit by linking lesion and cellular subtype to the mechanism most strongly implicated in this review and to a plausible therapeutic vulnerability, since most of the mechanistic sections above necessarily discuss “endometriotic lesions” as a single entity for narrative economy even though the underlying evidence is often subtype-specific.

Table 4 also clarifies how molecular subtype relates to three questions that matter clinically. First, on whether molecular differences track distinguishable lesion types: the evidence summarized above suggests they do, at least at the level of dominant mechanism—inflammatory and angiogenic in superficial peritoneal disease, estrogen- and hypoxia-driven in ovarian endometrioma, and TGF-β-driven and fibrotic in deep infiltrating disease—though these associations are drawn from cross-sectional tissue and expression studies rather than from prospective outcome data [33,58]. Second, on whether specific molecular signatures predict treatment response, this remains, as discussed in Section 10, a hypothesis rather than a validated finding, since no prospective study has yet tested whether baseline expression of the candidate microRNAs or other markers discussed above forecasts response to a specific therapy [14,15,41]. Third, on how molecular stratification could enter routine clinical decision-making, the most realistic near-term pathway is not upfront biomarker-based triage before any treatment decision, but stratification at the time of surgery, when tissue is already being obtained, using it to guide the choice of adjuvant or second-line therapy for patients whose disease persists or recurs after first-line hormonal treatment, an application that depends on the multi-omics and peritoneal fluid profiling approaches discussed in Section 10 reaching a validated, standardized assay form rather than remaining research-stage [4,54,55].

Beyond the modeling and heterogeneity problems above, the clinical feasibility of the emerging strategies discussed in Section 9 faces barriers that a mechanistic rationale alone does not address. Reproductive safety and fertility preservation are a first-order constraint rather than a secondary consideration in this population: anti-angiogenic agents validated in oncology carry a plausible risk to normal reproductive vasculature that has so far kept endometriosis-specific development at an early stage [5,18], and any strategy that suppresses ovulation, as most hormonal approaches do, is inherently difficult to reconcile with a treatment population in which a meaningful proportion of patients are actively trying to conceive. Long-term toxicity and systemic adverse effects are a second barrier: mTOR inhibitors and other oncology-derived agents carry a systemic side-effect burden that is easier to justify against a mortality risk than against a chronic, non-lethal condition managed over decades, which has so far limited enthusiasm for extended use of this drug class in endometriosis [7]. Achieving lesion-specific targeting compounds both problems: nucleic acid-based epigenetic therapies, for instance, are conceptually attractive precisely because they could act on a specific dysregulated microRNA or lncRNA rather than a pathway shared with healthy tissue, but delivering these agents selectively to peritoneal lesions rather than systemically remains an unresolved practical obstacle, and until it is resolved, the favorable selectivity these agents offer in principle cannot be realized in practice [38,39]. Taken together, these constraints mean that several of the mechanistically most attractive strategies in Table 2 are, at present, better described as candidates awaiting a solution to a delivery or selectivity problem than as therapies close to clinical testing.

Fertility considerations add a further real-world constraint largely absent from preclinical work: many patients are actively trying to conceive or wish to preserve that option, which rules out several of the more aggressive hormonal suppression strategies that might otherwise be tested, and complicates the design and interpretation of trials in a population where the desired treatment outcome, sustained ovulation suppression, is often directly at odds with a competing and equally important patient priority [51]. Finally, biomarker validation, revisited in Section 10, faces its own translational bottleneck: individual studies frequently report promising candidate markers, but the large, multi-center, prospectively designed cohorts needed to confirm reproducibility across diverse patient populations remain the exception rather than the rule in this field [42,53].

Figure 3 draws these threads together into a single integrative summary, connecting the lesion and cellular heterogeneity discussed throughout this review (Table 4) to the mechanistic axes covered in Section 2, Section 3, Section 4, Section 5, Section 6 and Section 7, the convergent signaling hubs detailed in Section 8, and the therapeutic and biomarker frameworks developed in Section 9 and Section 10, as a visual counterpart to the argument made in the Introduction.

## 12. Future Perspectives

Several directions seem likely to shape the next phase of endometriosis research and, eventually, care. Combination therapy that pairs hormonal suppression with an agent targeting a second axis, inflammatory, vascular, or metabolic, offers a more direct route to addressing the disease’s genuinely multi-mechanistic biology than any single-target approach reviewed in Section 9 is likely to achieve alone, and several of the candidate agents discussed there may find their most realistic clinical role in such combinations rather than as monotherapy [46,51]. Nanocarrier and targeted drug delivery platforms could meaningfully improve the practicality of several strategies discussed above, particularly the nucleic acid-based epigenetic therapies detailed in Section 9.4, whose clinical potential is currently constrained more by delivery limitations than by uncertainty about the underlying biological target [59]. Multi-omics integration, extending the tissue-based profiling introduced in Section 10 to larger and more diverse patient cohorts, offers the most plausible route toward the molecular subtyping this field increasingly recognizes as necessary, provided the field can also build the large-scale validation infrastructure that individual mechanistic studies have not, on their own, been able to supply [54,55]. Phenotype-guided treatment allocation, matching patients to therapy based on their dominant molecular subtype rather than uniform first-line hormonal suppression, represents less a specific technology than an organizing principle that the biomarker advances described above would need to make practically achievable [26]. Finally, continued development of non-hormonal therapeutic strategies, whether anti-inflammatory, anti-angiogenic, epigenetic, or metabolic in mechanism, matters specifically for the substantial population of patients for whom hormonal suppression is contraindicated, poorly tolerated, or simply insufficient, a group current treatment algorithms serve especially poorly. None of these directions offers a shortcut past the translational obstacles detailed in Section 11; what they offer, more modestly and more realistically, is a set of concrete next steps grounded in the multi-axis biology this review has described, rather than a return to treating endometriosis as a single hormonal disorder with a single hormonal solution [31,60].

## Figures and Tables

**Figure 1:**
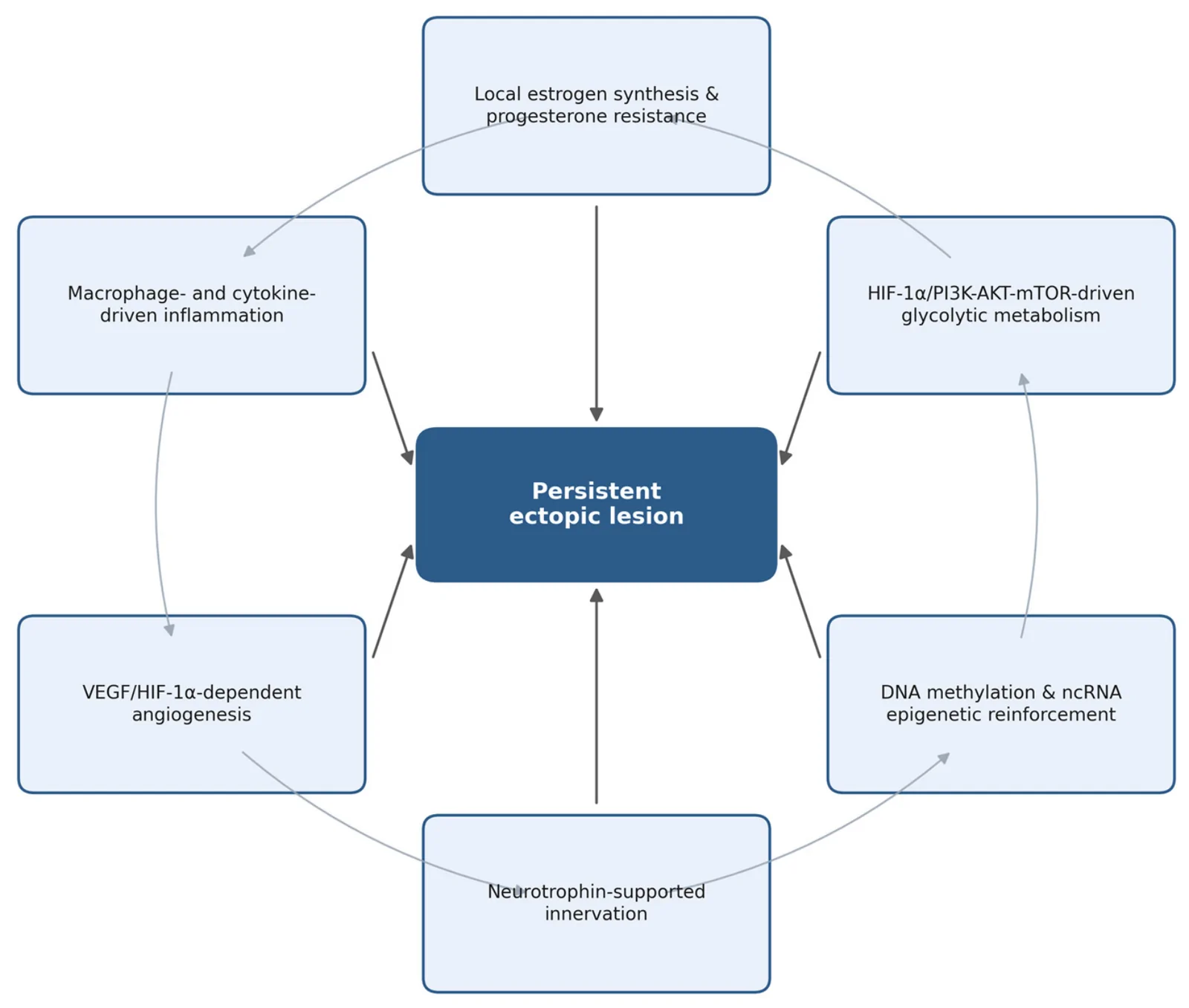
Overview of the interconnected processes sustaining endometriotic lesions, including local estrogen synthesis and progesterone resistance, chronic macrophage- and cytokine-driven inflammation, VEGF/HIF-1α-dependent angiogenesis, neurotrophin-supported innervation, DNA methylation and non-coding RNA-mediated epigenetic reinforcement, and HIF-1α/PI3K-AKT-mTOR-driven glycolytic metabolism. Arrows indicate the principal reinforcing relationships described in Section 2, Section 3, Section 4, Section 5, Section 6 and Section 7 of the text. Black arrows indicate a mechanism’s direct contribution to persistent lesion establishment; gray curved arrows indicate reinforcing relationships between adjacent mechanisms. The diagram is a qualitative synthesis of mechanisms reported across independent studies rather than a quantitative or validated causal model; arrow direction indicates a reported reinforcing relationship, not its relative magnitude, and the causal hierarchy among these processes has not been established and may vary by lesion subtype.

**Figure 2:**
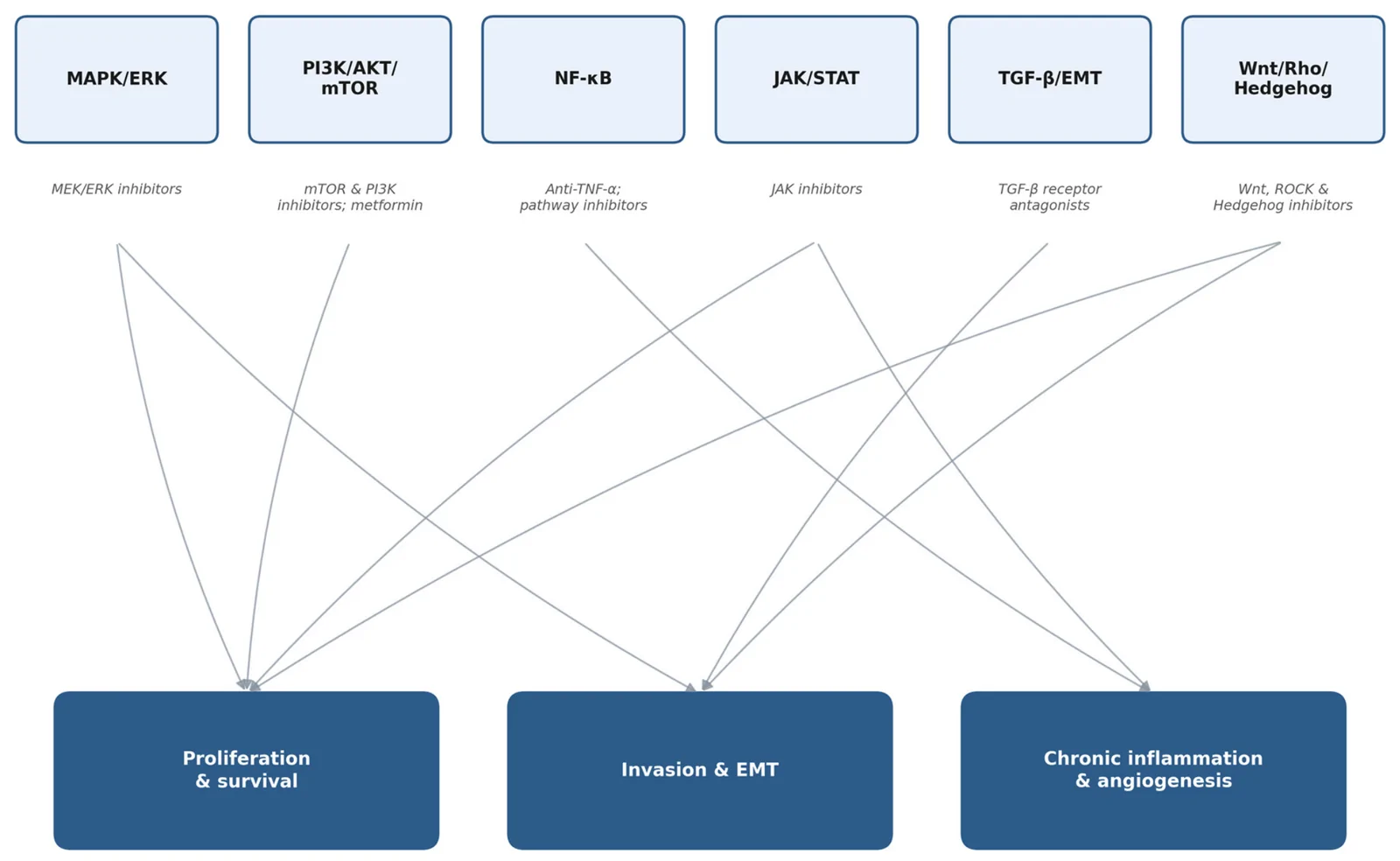
Molecular pathway map indicating points of convergence among MAPK/ERK, PI3K/AKT/mTOR, NF-κB, JAK/STAT, TGF-β/EMT, and Wnt/Rho/Hedgehog signaling, annotated with the candidate therapeutic targets discussed in Section 9. Pathway placement in this map does not imply equivalent validation; the evidence tier underlying each pathway is defined separately in Table 1 and should be consulted before inferring clinical readiness from this figure alone.

**Figure 3:**
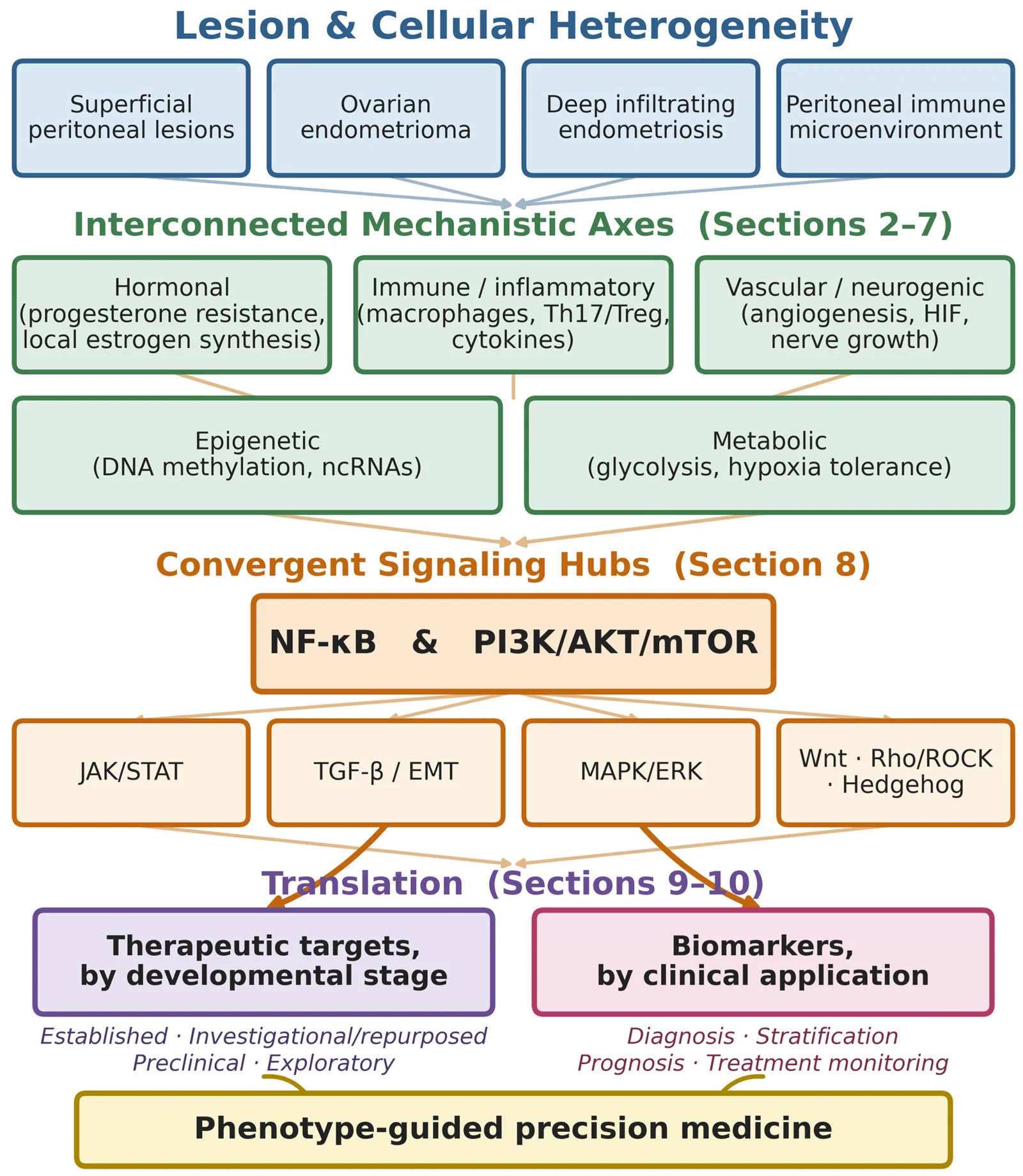
Integrative summary linking lesion and cellular heterogeneity (Table 4) to the interconnected mechanistic axes of Section 2, Section 3, Section 4, Section 5, Section 6 and Section 7, the convergent signaling hubs of Section 8, and the therapeutic (Section 9, Table 2) and biomarker (Section 10, Table 3) frameworks that together define the phenotype-guided precision medicine approach argued for throughout this review.

**Table 1:** Molecular pathways, their principal biological function in endometriosis, candidate therapeutic targets, and supporting evidence level.

Pathway	Principal Biological Function	Candidate Therapeutic Target/Approach	Evidence Level (Tier)
MAPK/ERK	Proliferation, migration, survival; MMP-dependent invasion	Upstream growth factor receptors; MEK/ERK inhibitors (oncology-derived)	Tier II. Human tissue and in vitro studies; no endometriosis-specific clinical trials
PI3K/AKT/mTOR	Growth factor/cytokine-driven survival, glycolytic metabolism, apoptosis resistance	mTOR inhibitors; PI3K inhibitors; metformin (AMPK-linked)	Tier II (Tier I for metformin repurposing). Human tissue, in vitro, and rodent models; early-phase repurposing trials (metformin)
NF-κB	Central inflammatory transcription hub; cytokine, adhesion molecule and angiogenic gene expression	Pathway inhibitors; upstream cytokine blockade (e.g., anti-TNF-α)	Tier II. Human tissue, in vitro, and rodent models; no pathway-directed endometriosis trials
JAK/STAT	Propagation of IL-6-driven chronic inflammation; survival signaling	JAK inhibitors (repurposed from autoimmune disease)	Tier II. Human tissue and in vitro studies; JAK inhibitor trials exist only in other inflammatory diseases
TGF-β/EMT signaling	Fibrogenesis; EMT-like invasive phenotype, prominent in deep infiltrating disease	TGF-β receptor antagonists (preclinical)	Tier II. Human tissue, in vitro, and rodent models; preclinical stage only
Wnt/β-catenin	Proliferation; acquisition of mesenchymal, invasive phenotype	Wnt pathway inhibitors (early preclinical)	Tier III. In vitro studies with limited human tissue confirmation; preclinical stage only
Rho/ROCK	Cytoskeletal remodeling supporting cell migration and invasion	ROCK inhibitors (preclinical)	Tier III. In vitro and rodent models; preclinical stage only
Hedgehog	Elevated in ectopic lesions; functional contribution less well defined	Hedgehog pathway inhibitors (exploratory)	Tier IV. Human tissue (elevated expression only); exploratory, no functional or interventional studies

**Table 2:** Current and experimental therapeutic strategies, classified by developmental stage, evidence level, expected benefit, safety concern, and target patient population.

Category	Representative Agent/Approach	Developmental Stage	Evidence Level	Principal Expected Benefit	Major Safety Concern	Target Patient Population
Hormonal	Progestins	Established	Multiple RCTs; clinical guidelines	Reliable symptom control	Breakthrough bleeding; limited efficacy with PR resistance	Broad; first-line
Hormonal	GnRH agonists/antagonists (e.g., elagolix, relugolix)	Established	Multiple pivotal RCTs; regulatory approval	Pain reduction; antagonists avoid initial flare	Hypoestrogenic effects; bone density loss with prolonged use	Patients failing progestins; add-back therapy often needed
Hormonal	Aromatase inhibitors	Established (refractory-line)	Trials in refractory disease	Targets local estrogen synthesis	Bone loss, joint pain; not for monotherapy premenopausally	Refractory cases only
Hormonal	Selective progesterone receptor modulators	Investigational in endometriosis (established in fibroids)	Mature evidence in fibroids; limited endometriosis-specific trials	Engages residual PR activity	Endometrial changes with long-term use	Patients with marked PR-A/PR-B imbalance
Anti-inflammatory	Anti-TNF-α agents	Tested, discontinued	Completed clinical trials; negative results	None demonstrated	—	Approach largely abandoned
Anti-inflammatory	COX-2 inhibitors	Established (symptomatic use)	Clinical use; cardiovascular safety data	Pain relief	Cardiovascular risk with long-term use	Short-term symptom control only
Anti-inflammatory	NF-κB/JAK-STAT-directed strategies	Investigational (repurposed)	No endometriosis-specific trials	Broad anti-inflammatory effect	Systemic immunosuppression risk	Not yet defined
Anti-angiogenic	VEGF-targeted agents	Preclinical/early investigational	Validated in oncology; not in endometriosis	Disrupts lesion vascularization	Risk to reproductive tissue and fertility	Excludes patients seeking fertility preservation
Anti-neurogenic	NGF-pathway-directed approaches	Preclinical	Rodent and in vitro data	Interrupts pain independent of lesion size	Not established	Patients with pain-lesion burden mismatch
Epigenetic	HDAC inhibitors	Preclinical	In vitro and rodent models	Reverses chromatin dysregulation	Class-wide toxicity (oncology experience)	Not yet defined
Epigenetic	microRNA mimics/antagomirs	Preclinical	In vitro studies	Targets specific epigenetic lesions	Peritoneal delivery unresolved	Not yet defined
Metabolic	mTOR inhibitors	Preclinical (oncology-derived)	In vitro and rodent models	Addresses glycolytic/proliferative phenotype	Systemic side-effect burden	Not favored for long-term use in benign disease
Metabolic	Metformin	Investigational (repurposed)	Limited endometriosis data; extensive safety data elsewhere	Favorable safety; mild anti-inflammatory effect	Modest expected effect size	Broad; good long-term safety profile
Metabolic	Antioxidant strategies	Exploratory/adjunctive	Modest, inconsistent trial results	Adjunctive oxidative stress reduction	Low	Adjunct only, not primary therapy
Immunomodulatory/precision	Immune-profile-guided stratification	Conceptual framework	No validated clinical tool yet	Matches therapy to immune phenotype	Depends on unvalidated biomarkers	Not yet actionable

**Table 3:** Candidate biomarkers, organized by primary clinical application (diagnosis, stratification, or prognosis) rather than sample source alone, with current validation status.

Biomarker Category	Sample Source	Primary Clinical Application	Validation Status
CA-125	Serum	Diagnosis (adjunct only)	Widely used clinically but insufficiently sensitive/specific as a standalone diagnostic; not recommended alone
Inflammatory cytokine panels	Serum/plasma	Diagnosis; possible severity correlation	Modest cross-study reproducibility; not externally validated
Circulating microRNAs	Serum/plasma	Diagnosis (research stage); potential treatment-response prediction	Not recommended for clinical diagnosis outside research [52]; reproducibility limited by pre-analytical and analytical variability
NF-κB (PBMC p65 expression)	Peripheral blood	Prognosis (recurrence prediction)	Preliminary cohorts only; no standardized assay
Peritoneal fluid cytokine/immune profiling	Peritoneal fluid	Stratification and mechanistic characterization at time of surgery	Consistent mechanistic findings; not suited to non-invasive diagnosis by definition
Tissue/lesion multi-omics profiling	Lesion biopsy	Stratification; research-stage precision medicine framework	Research-stage; lacks large, prospectively validated cohorts

**Table 4:** Endometriosis lesion and cellular subtypes, their predominant molecular mechanism as discussed in this review, and potential therapeutic vulnerability.

Lesion/Cellular Subtype	Predominant Molecular Mechanism (This Review)	Potential Therapeutic Vulnerability
Superficial peritoneal lesions	Inflammatory and immune-evasion mechanisms dominant; active angiogenesis and neurogenesis; comparatively limited fibrosis (Section 4 and Section 5)	Anti-inflammatory and anti-angiogenic strategies; NGF-pathway approaches for pain
Ovarian endometrioma	Local estrogen synthesis and progesterone resistance prominent; hypoxia-adapted glycolytic phenotype and cyst-fluid oxidative stress (Section 3 and Section 7)	Aromatase inhibition; metabolic and antioxidant-targeted approaches
Deep infiltrating endometriosis	TGF-β-driven fibrogenesis and EMT-like invasive phenotype dominant (Section 5 and Section 8.5)	TGF-β pathway antagonists (preclinical); surgical excision remains primary treatment
Eutopic endometrium (in affected women)	HOXA10 hypermethylation and altered receptivity gene expression (Section 6)	Relevant mainly to infertility-focused management rather than lesion-directed therapy
Ectopic stromal cells	Aromatase overexpression, PR-A/PR-B imbalance, NF-κB activation (Section 3, Section 4 and Section 8.3)	Hormonal and NF-κB-directed strategies
Ectopic epithelial cells	EMT-like transition, MAPK/ERK-driven, MMP-dependent invasion (Section 2, Section 8.1 and Section 8.5)	MAPK/ERK and TGF-β-directed approaches (preclinical)
Peritoneal immune microenvironment	Macrophage polarization (JAK/STAT6), Th17/Treg imbalance, NK-cell dysfunction (Section 4)	Immune-profile-guided stratification; immunomodulatory approaches (conceptual)

## Data Availability

No new data were created or analyzed in this study. Data sharing is not applicable to this article.

## Abbreviations

AMPK	AMP-activated Protein Kinase
BCCIP	BRCA2 and CDKN1A-Interacting Protein
BCL-2	B-cell Lymphoma 2
BPA	Bisphenol A
CA-125	Cancer Antigen 125
CDK6	Cyclin-Dependent Kinase 6
circRNA	Circular RNA
COX-2	Cyclooxygenase-2
EDC	Endocrine-Disrupting Chemical
EMT	Epithelial–Mesenchymal Transition
ERβ	Estrogen Receptor beta
ERK	Extracellular signal-Regulated Kinase
Foxp3	Forkhead Box P3
GLUT1	Glucose Transporter 1
GnRH	Gonadotropin-Releasing Hormone
GWAS	Genome-Wide Association Study
HDAC	Histone Deacetylase
HIF-1α	Hypoxia-Inducible Factor 1-alpha
HIF-2α	Hypoxia-Inducible Factor 2-alpha
HIF-3α	Hypoxia-Inducible Factor 3-alpha
HOTAIR	HOX Transcript Antisense RNA
HOXA10	Homeobox A10
IKK	IκB Kinase
IL-1β	Interleukin 1 beta
IL-6	Interleukin 6
JAK/STAT	Janus Kinase/Signal Transducer and Activator of Transcription
lncRNA	Long Non-Coding RNA
MALAT1	Metastasis-Associated Lung Adenocarcinoma Transcript 1
MAPK/ERK	Mitogen-Activated Protein Kinase/Extracellular signal-Regulated Kinase
MCP-1	Monocyte Chemoattractant Protein-1
MEK	Mitogen-activated protein kinase Kinase
miRNA	MicroRNA
MMP	Matrix Metalloproteinase
mTOR	Mechanistic Target of Rapamycin
ncRNA	Non-Coding RNA
NF-κB	Nuclear Factor kappa-light-chain-enhancer of activated B cells
NGF	Nerve Growth Factor
PGE2	Prostaglandin E2
PI3K/AKT/mTOR	Phosphoinositide 3-Kinase/Protein Kinase B/Mechanistic Target of Rapamycin
PR-A/PR-B	Progesterone Receptor isoform A/isoform B
Rho/ROCK	Ras Homolog Family Member/Rho-associated Coiled-coil containing protein Kinase
RORγt	RAR-related Orphan Receptor gamma t
TCDD	2,3,7,8-Tetrachlorodibenzo-p-dioxin
TCF7L1/TCF7L2	Transcription Factor 7-Like 1/2
TGF-β	Transforming Growth Factor beta
Th17	T helper 17 (cell)
TNF-α	Tumor Necrosis Factor alpha
VEGF(-A)	Vascular Endothelial Growth Factor (-A)
Wnt	Wingless/Integrated (signaling pathway)
ZEB1/ZEB2	Zinc finger E-box-Binding homeobox 1/2
